# Cardioprotection by post-conditioning with exogenous triiodothyronine in isolated perfused rat hearts and isolated adult rat cardiomyocytes

**DOI:** 10.1007/s00395-021-00868-6

**Published:** 2021-04-19

**Authors:** Helmut Raphael Lieder, Felix Braczko, Nilgün Gedik, Merlin Stroetges, Gerd Heusch, Petra Kleinbongard

**Affiliations:** Institute for Pathophysiology, West German Heart and Vascular Center, University of Essen Medical School, Hufelandstr. 55, 45122 Essen, Germany

**Keywords:** Cardioprotection, Ischemia/reperfusion, Ischemic conditioning, Post-conditioning, Triiodothyronine

## Abstract

Ischemic post-conditioning (iPoCo) by coronary re-occlusion/reperfusion during immediate reperfusion after prolonged myocardial ischemia reduces infarct size. Mechanical manipulation of culprit lesions, however, carries the risk of coronary microembolization which may obscure iPoCo’s cardioprotection. Pharmacological post-conditioning with exogenous triiodothyronine (T3) could serve as an alternative conditioning strategy. Similar to iPoCo, T3 may activate cardioprotective prosurvival pathways. We aimed to study T3’s impact on infarct size and its underlying signal transduction. Hearts were isolated from male Lewis rats (200–380 g), buffer-perfused and subjected to 30 min/120 min global zero-flow ischemia/reperfusion (I/R). In additional hearts, either iPoCo (2 × 30 s/30 s I/R) was performed or T3 (100–500 µg/L) infused at reperfusion. Infarct size was demarcated with triphenyl tetrazolium chloride staining and calculated as percent of ventricular mass. Infarct size was reduced with iPoCo to 16 ± 7% vs. 36 ± 4% with I/R only. The maximum infarct size reduction was observed with 300 µg/L T3 (14 ± 2%). T3 increased the phosphorylation of protein kinase B and mitogen extracellular-regulated-kinase 1/2, both key enzymes of the reperfusion injury salvage kinase (RISK) pathway. Pharmacological RISK blockade (RISK-BL) during reperfusion abrogated T3’s cardioprotection (35 ± 10%). Adult ventricular cardiomyocytes were isolated from buffer-perfused rat hearts and exposed to 30 min/5 min hypoxia/reoxygenation (H/R); reoxygenation was initiated without or with T3, respectively, and without or with RISK-BL, respectively. Maximal preservation of viability was observed with 500 µg/L T3 after H/R (27 ± 4% of all cells vs. 5 ± 3% in time-matched controls). Again, RISK-BL abrogated protection (11 ± 3%). Mitochondria were isolated at early reperfusion from buffer-perfused rat hearts without or with iPoCo or 300 µg/L T3, respectively, at reperfusion. T3 improved mitochondrial function (i.e.: increased respiration, adenosine triphosphate production, calcium retention capacity, and decreased reactive oxygen species formation) to a similar extent as iPoCo. T3 at reperfusion reduces infarct size by activation of the RISK pathway. T3’s protection is a cardiomyocyte phenomenon and targets mitochondria.

**Supplementary Information:**

The online version contains supplementary material available at 10.1007/s00395-021-00868-6.

## Introduction

In patients with acute myocardial infarction, the only way to salvage myocardium at risk is early reperfusion. Despite the successful implementation and use of percutaneous coronary interventions (PCI) in patients with acute ST-segment elevation myocardial infarction, their 1-year mortality remains at ~ 15% as reported in a recent large registry [65]. Therefore, there is still an unmet need to protect myocardium at risk of infarction beyond the protection induced by early reperfusion [23]. Myocardial damage is not only determined by ischemic, but also by reperfusion injury [6, 27, 67]. Cycles of coronary re-occlusion/reperfusion at early reperfusion (ischemic post-conditioning; iPoCo) attenuate ischemia/reperfusion (I/R) injury and reduce infarct size in preclinical and clinical studies [19, 51, 64]. In patients with acute myocardial infarction, iPoCo is induced during primary PCI by repetitive short re-occlusions using an angioplasty balloon after initial re-opening of the occluded coronary artery [25, 64]. However, the mechanical manipulation of culprit lesions carries the risk of coronary microembolization with subsequent injury [30], and iPoCo requires mechanical manipulation. Such iatrogenic microembolization may obscure iPoCo’s cardioprotection [22]. Thus, pharmacological post-conditioning appears as an attractive alternative or additive strategy to induce cardioprotection [9]. In experimental models of myocardial infarction [12, 52], but also in patients with acute ST-segment elevation myocardial infarction [57], exogenous triiodothyronine (T3) infusion during reperfusion restored the reduced endogenous T3 levels and improved left ventricular (LV) contractile function. T3 is the most important endogenous biologically active thyroid hormone, and T3 replacement therapy aims to restore physiological T3 levels [16]. T3 in doses which result in supraphysiological plasma T3 levels during reperfusion also improved the recovery of coronary flow (CF) [4] and LV contractile function [53] in isolated perfused rat hearts with global I/R. Recovery of LV contractile function is certainly a clinically relevant endpoint, but mechanistically difficult to interpret, as it reflects loss of viable tissue (infarct size), the time-dependent recovery of reversibly injured myocardium (stunning) [28], and the potentially adaptive contractile function of remote myocardium. Thus, infarct size is a more robust endpoint to study cardioprotection [5, 42]. Whether or not acute T3 treatment only improves LV contractile function or also reduces infarct size is currently unknown. T3 induces multiple genomic and non-genomic effects in the heart and vascular system [32, 56, 60], and the acute T3-induced effects must primarily rely on non-genomic effects.

Several intracellular survival pathways have been identified in the context of cardioprotection and conceptually categorized as the nitric oxide synthase (NOS)/protein kinase G pathway, the reperfusion injury salvage kinase (RISK) pathway, and the survival activating factor enhancement (SAFE) pathway [27]. These pathways transmit their cardioprotective signal to end-effectors, notably the mitochondria [8]. Phosphatidylinositol(4,5)-bisphosphate-3-kinase/protein kinase B (PI3K/AKT) [50] and mitogen extracellular-regulated kinases (ERK)1/2 are key enzymes of the RISK pathway [24, 61]. Downstream of RISK activation, the phosphorylation and thus inhibition of glycogen synthase kinase 3β (GSK-3β) was proposed to inhibit mitochondrial permeability transition pore (mPTP) opening, and thus to mediate cardioprotection [34]. In rats, with permanent left anterior descending coronary artery occlusion, chronic T3 and thyroxine treatment over 12 days starting at day three after occlusion resulted in a decreased myocardial AKT expression and an increased AKT phosphorylation [50]. Also, in isolated rat aortic vascular smooth muscle cells, acute T3 treatment activated PI3K/AKT [7]. In the myocardium, however, none of the classical cardioprotective pathways has yet been reported to be activated by acute T3 treatment. T3 interacts with membrane integrin receptors, which mediate the activation of ERK1/2 [3, 47]. Therefore, T3 is hypothesized to activate the classical cardioprotective RISK pathway. T3 also targets mitochondria: T3 given during reperfusion in isolated perfused rat hearts re-inforced mitophagy through a phosphatase tensin homolog-induced kinase 1/parkin-dependent mechanism [4, 11].

We have now studied the impact of exogenous T3, given at reperfusion, on infarct size in isolated buffer-perfused rat hearts subjected to global zero-flow I/R. In rat myocardium, cardioprotection by ischemic and pharmacological conditioning activates RISK and/or SAFE pathways [18, 21, 43, 58, 59]. We therefore focused on these pathways and on GSK-3β as a downstream target of the RISK pathway and analyzed them in myocardial tissue samples. To identify cardioprotection at the cardiomyocyte level, isolated adult rat ventricular cardiomyocytes were subjected to hypoxia/reoxygenation (H/R) without or with T3 during reoxygenation. The causal involvement of RISK and/or SAFE activation was addressed by use of pharmacological blockers. The impact of T3 on mitochondrial respiration, adenosine triphosphate (ATP) production, reactive oxygen species (ROS) formation and calcium retention capacity (CRC) as potential effectors of cardioprotection was also assessed. We used iPoCo as a reference for the cardioprotection by T3.

## Methods

All data of the present exploratory study are available in the article and its Online Resources. Experiments were performed between December 2019 and December 2020 using contemporary block randomization. We have recently shown that in our rat model sex has no impact on the cardioprotection by ischemic conditioning [39]. Therefore, only male Lewis rats (200–380 g, 2.0–3.5 months, Central Animal Laboratory, University of Duisburg-Essen, Essen, Germany) were used in the present study. The experimental protocols in isolated buffer-perfused rat hearts, cardiomyocytes and mitochondria as well as the methods for the measurement of hemodynamics and quantification of infarct size and cardiomyocyte viability were standard [5, 42] and have been described in detail previously [15, 40, 63]. For details, see Online Resources. Unless otherwise specified, materials were obtained from Sigma-Aldrich (Deisenhofen, Germany).

### Isolated buffer-perfused hearts

To study whether or not T3 reduces infarct size, isolated buffer-perfused rat hearts were subjected to I/R. Hearts were reperfused with Krebs–Henseleit buffer. Exogenous T3 was added in increasing concentrations (100, 200, 300, 500 µg/L) to determine the T3 concentration which maximally reduced infarct size. This T3 concentration (300 µg/L) was then used in the following experiments to analyze the potential activation of cardioprotective signaling pathways: to block RISK activation (RISK-BL) the PI3K blocker wortmannin (1 µmol/L) and the mitogen extracellular-regulated-kinase (ERK)1/2 blocker U0126 (1 µmol/L) were added to the perfusion buffer. To distinguish between activation of the RISK pathway kinases PI3K and ERK1/2, experiments with T3 at reperfusion were repeated under either PI3K blockade (PI3K-BL) or ERK1/2 blockade (ERK-BL). The signal transducer and activator of transcription (STAT)3 blocker stattic (1 µmol/L) was used to block SAFE pathway activation (SAFE-BL). T3 was dissolved in NaOH (40 mmol/L, final dilution of at maximum 1:10,000 in the perfusion buffer). Blockers were dissolved in dimethylsulfoxide (final dilution in the perfusion puffer 1:10,000). The blocker concentrations have been previously established in a comparable experimental setup; RISK-BL abrogated the increased phosphorylation of AKT1/2/3_Ser473_ and ERK1/2_Thr202–Tyr204/Thr185–Tyr187_ induced by cardioprotective maneuvers in rat myocardium [62, 63]. The solvents NaOH and dimethylsulfoxide per se had no impact on infarct size. T3’s cardioprotection was compared to that induced by iPoCo.

#### Experimental preparation and protocols

Rats were sacrificed, their hearts isolated, immediately mounted on a Langendorff-apparatus and perfused with modified Krebs–Henseleit buffer at constant pressure of 65–70 mmHg. CF and LV developed pressure (LVDP) were continuously recorded, and heart rate was kept at 360 beats per min by right atrial pacing [38]. After completion of reperfusion, myocardial biopsies were taken from the heart’s apex (~ 10–20 mg) and quickly frozen in liquid nitrogen for later protein analysis by western blot. Infarct size was demarcated by triphenyl tetrazolium chloride staining and calculated as percent of the sum of left and right ventricular mass (% of ventricular mass). For details, see Online Resources, material section.

**Fig. 1:**
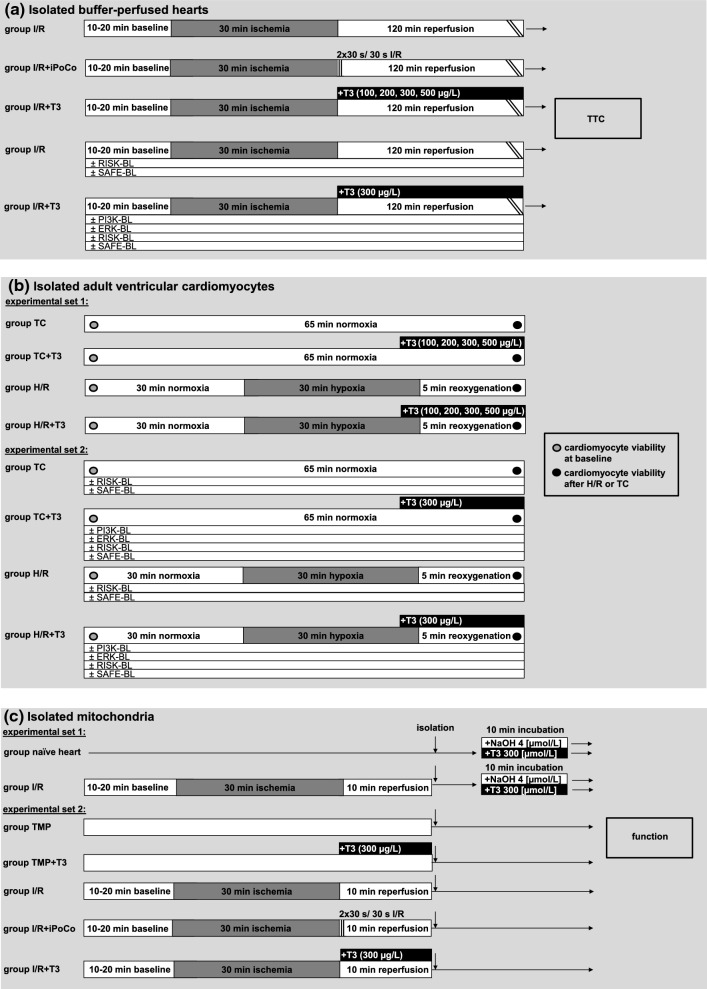
Experimental groups and protocols. *ERK-BL* mitogen extracellular-regulated-kinase phosphorylation blockade, *H/R* hypoxia and reoxygenation, *iPoCo* ischemic post-conditioning, *I/R* ischemia and reperfusion, *PI3K-BL* phosphatidylinositol(4,5)-bisphosphate-3-kinase blockade, *RISK-BL* reperfusion injury salvage kinase pathway blockade, *SAFE-BL* survival activating factor enhancement pathway blockade, *T3* triiodothyronine, *TC* time control, *TMP* time-matched perfusion, *TTC* infarct size demarcation by triphenyl tetrazolium chloride staining

Preparations were allowed to stabilize for 10–20 min, before baseline values for CF and LVDP were recorded. Block randomization with sealed envelopes was then used for the allocation of isolated buffer-perfused hearts to the following groups (Fig. 1a):

I/R: Ischemia was induced globally by stopping coronary flow for 30 min followed by 120 min reperfusion, prior to measurement of infarct size (*n* = 10).

I/R + iPoCo: After 30 min ischemia, iPoCo was induced 30 s after the onset of reperfusion by two cycles of 30 s full stop of perfusion and 30 s reperfusion, and reperfusion was then continued for 118 min (*n* = 15).

I/R + T3: After 30 min ischemia, the perfusion buffer was switched to modified Krebs–Henseleit buffer with T3 (100, *n* = 12; 200, *n* = 13; 300, *n* = 12; 500, *n* = 11; in µg/L). Experiments with blockers of the RISK and SAFE pathway (I/R + T3 + PI3K-BL, *n* = 10; I/R + T3 + ERK-BL, *n* = 10; I/R + T3 + RISK-BL, *n* = 9; I/R + T3 + SAFE-BL, *n* = 10), respectively, were performed with 300 µg/L T3, because this T3 concentration induced the maximal infarct size reduction.

#### Phosphorylation of cardioprotective proteins

Protein phosphorylation was analyzed in biopsies taken from the isolated buffer-perfused hearts after 120 min reperfusion. From hearts with I/R, I/R + iPoCo or I/R + T3 (300 µg/L), *n* = 8 myocardial tissue biopsies, respectively, were randomly selected to have them all analyzed on the same gel/membrane (Online Resources, Online Fig. 1). Myocardial tissue biopsies were homogenized and protein aliquots were electrophoretically separated on precast stain-free 12% (for AKT1/23, ERK1/2 and STAT3) or 7.5% (for GSK-3β) sodium dodecyl sulfate polyacrylamide electrophoresis gels (BioRad, Hercules, USA). In preliminary experiments, for each analyzed protein and its phosphorylated form, the combined linear range had been determined according to the manufacturer’s protocol [55], and the respective protein quantity within the linear range was then used for western blotting. Total protein fluorescence was activated by ultraviolet light exposition, imaged (Gel Doc EZ system, Bio Rad) and visually examined for equal loading. Proteins were transferred to 0.45 µm low fluorescence polyvinylidene difluoride membranes (Merck, Chemicals GmbH, Darmstadt, Germany). Membranes were cut into three parts and then incubated with primary antibodies directed against the phosphorylated forms of AKT1/2/3_Ser473_, ERK1/2_Thr202-Tyr204/Thr185-Tyr187_, GSK-3β_Ser9_, or STAT3_Tyr705_. Membranes were then incubated with the respective antibodies directed against the total forms of AKT1/2/3, ERK1/2, GSK-3β or STAT3, respectively, before adding the secondary antibodies. Fluorescence signal intensity was imaged using the LI-COR Biosciences infrared imaging system (LI-COR Biosciences, Lincoln, USA). Detected signals were analyzed with the LI-COR Biosciences Empiria^®^ studio software (version 1.3.0.83). Fluorescence signal intensity of each phosphorylated protein was normalized to the respective signal of the total protein form. For details, see Online Resources.

### Isolated adult ventricular cardiomyocytes

To identify whether or not the protection by exogenous T3 is a cardiomyocyte phenomenon, rats were sacrificed, hearts buffer-perfused, and adult ventricular cardiomyocytes isolated by enzymatic digestion. Cardiomyocytes were isolated and kept in normoxic buffer for 5 min before viability was determined at baseline. Experimental set 1 (Fig. 1b): cardiomyocytes from each heart were divided into H/R and time control (TC) groups. Hypoxia was induced for 30 min by exposing cardiomyocytes to hypoxic, glucose-free buffer, pH adjusted to 6.5, and sealing with mineral oil; cells were kept in solution where they sediment; reoxygenation was induced by removal of oil and hypoxic buffer and adding of reoxygenation buffer for 5 min. In TC experiments, cardiomyocytes were exposed to normoxic buffer for 65 min. The viability of cardiomyocytes was quantified after 5 min (baseline) and at 65 min after H/R and TC, respectively, in all groups and expressed as the percentage of rod-shaped, trypan blue-negative cardiomyocytes over the total number of cells. To evaluate the impact of T3 on cardiomyocyte viability, T3 (100, 200, 300, 500 µg/L) was added to the hypoxia buffer at 25 min hypoxia and to the reoxygenation buffer, and in TC groups to normoxic buffers, respectively. Experiments with different T3 concentrations were performed to determine the T3 concentration that maximally preserved viability. This T3 concentration (500 µg/L) was then used for subsequent blocker experiments in experimental set 2: again, cardiomyocytes from each heart were divided into H/R and TC groups. Cardiomyocytes were incubated with T3 (500 µg/L) without or with PI3K-BL, ERK-BL, RISK-BL or SAFE-BL, respectively. Blockers were added to the buffers throughout the whole experiment. T3 and blockers were dissolved and used in the same concentrations as described above. NaOH and dimethylsulfoxide per se had no impact on cardiomyocyte viability. For details, see Online Resources.

### Isolated mitochondria

To verify whether or not there is a direct effect of T3 on mitochondria, in experimental set 1 (Fig. 1c) mitochondria were isolated from naïve hearts or buffer-perfused hearts with I/R and incubated with 300 µg/L T3 or 4 µmol/L NaOH, respectively, in vitro for 10 min. Mitochondrial adenosine diphosphate (ADP, 0.4 mmol/L)-stimulated complex I respiration, complex IV respiration after addition of *N*,*N*,*N*,*N*′-tetramethyl-*p*-phenylenediamine (TMPD, 300 μmol/L) and ascorbate (3 mmol/L) and maximal uncoupled oxygen uptake in the presence of carbonyl cyanide-*p*-trifluoro-methoxyphenyl-hydrazone (FCCP, 30 nmol/L) were measured with a Clark type electrode (Strathkelvin, Glasgow, UK). Mitochondrial ATP production was measured using ATP assay mix and compared to ATP standards by spectrometry (F-7100, Hitachi High-Tech, Krefeld, Germany). Mitochondrial ROS formation was measured using the amplex™ red hydrogen peroxide assay (Thermo Fisher Scientific, Waltham, USA). CRC was determined using glutamate/malate as substrates in the presence of ADP. Calcium green™-5N (Thermo Fisher Scientific) was used to measure the extramitochondrial calcium concentration in a spectrophometer. Pulses of CaCl_2_ (5 nmol/L) were added (1/min) until a rapid increase in calcium green fluorescence indicated mPTP opening [15]. Cyclosporine A delays mPTP opening by interaction with cyclophilin D to keep the pore closed. Therefore, additional measurements with cyclosporine A (10 µmol/L) served as a positive control [1, 15].

In experimental set 2 (Fig. 1c) the protocols as in groups I/R, I/R + iPoCo, and I/R + T3 (300 µg/L) were repeated (*n* = 8, each), and reperfusion was stopped after 10 min. For the respective control experiments, hearts were perfused for additional 30 min after baseline values for CF and LVDP had been recorded, followed either by 10 min perfusion without (time-matched perfusion, TMP, *n* = 8) or with 300 µg/L T3 (TMP + T3, *n* = 8). Mitochondria were isolated at 10 min reperfusion or at the corresponding time point in TMP experiments, respectively, before analysis of mitochondrial function, as described above. For details, see Online Resources.

### Statistics

Investigators performing experiments in isolated buffer-perfused hearts, cardiomyocytes, and mitochondria and analyzing infarct size and time courses of CF and LVDP in isolated buffer-perfused hearts, cardiomyocyte viability and mitochondrial respiration, ATP production, ROS formation and CRC were blinded with respect to group assignment and treatment. Investigators analyzing data sets were blinded with respect to the protocols. Investigators who performed iPoCo and investigators who analyzed CF and LVDP in isolated buffer-perfused hearts with iPoCo could not be blinded, since iPoCo impacts on CF and LVDP. The Kolmogorov–Smirnov test was used to test normality for all data sets. The assumption of normality was confirmed for all analyzed data sets, except for the protein fluorescence signal intensity of GSK-3β. Data are presented as means ± standard deviations or as median [interquartile range]. Time courses of CF and LVDP in isolated buffer-perfused hearts were analyzed by two-way (time, group) ANOVA for repeated measures. One-way ANOVA was used to analyze CF and LVDP in isolated buffer-perfused hearts at baseline, infarct size in isolated buffer-perfused rat hearts, viability in isolated cardiomyocytes, mitochondrial respiration, ATP production and ROS formation in mitochondria and fluorescence signal intensity of protein phosphorylation (AKT1/2/3_Ser473_, ERK1/2_Thr202-Tyr204/Thr185-Tyr187_, STAT3_Tyr705_) in myocardial biopsies. Individual mean values of data sets were compared by Fisher’s least-significant-difference post-hoc tests when ANOVA indicated a significant difference (SigmaStat 3.5, Erkrath, Germany). One-way Kruskal–Wallis ANOVA on ranks with Tukey’s multiple comparisons procedures was used to analyze the signal intensity of GSK-3β phosphorylation. Differences were considered significant at the level of *p* < 0.05, and exact *p* values are given for *p* values when ≥ 0.01 for infarct size, cardiomyocyte viability, functional parameters of isolated mitochondria and fluorescence signal intensity of phosphorylated AKT1/2/3_Ser473_, ERK1/2_Thr202-Tyr204/Thr185-Tyr187_, GSK-3β_Ser9_ and STAT3_Tyr705_.

## Results

### Coronary flow, left ventricular function, infarct size and cardioprotective proteins

**Fig. 2:**
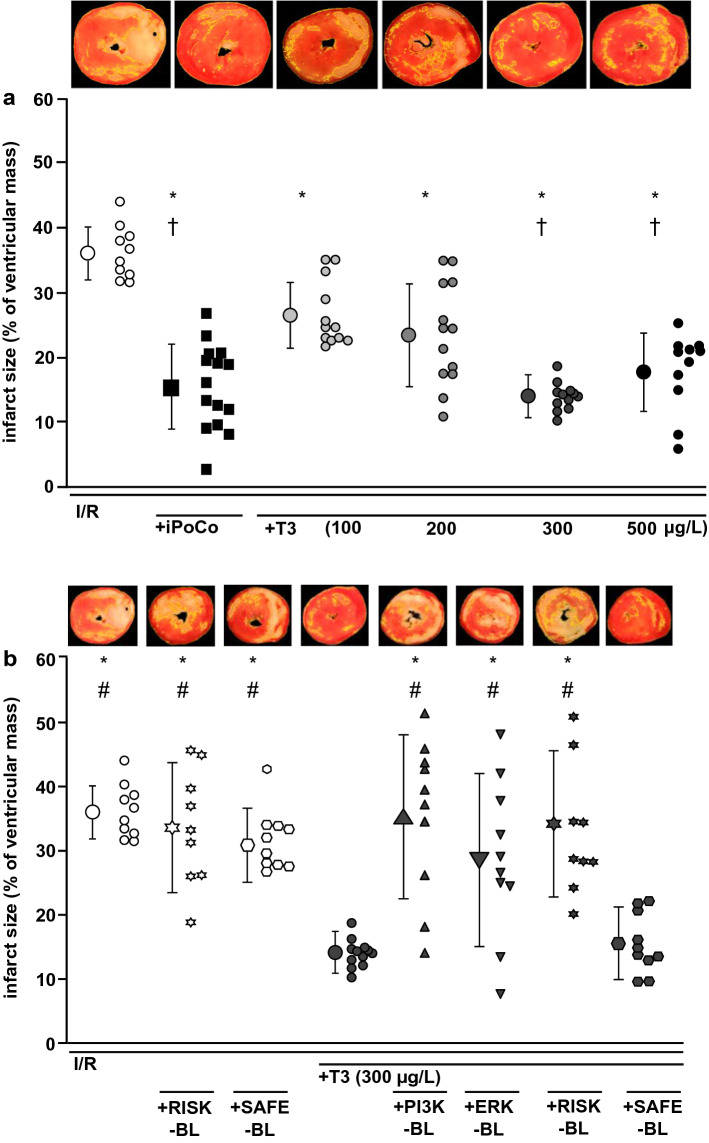
Impact of T3 at reperfusion on infarct size in isolated buffer-perfused rat hearts. Data are presented as means ± standard deviations. For each group, representative triphenyl tetrazolium chloride-stained heart slices are displayed; areas enclosed by yellow lines indicate infarcted tissue. *ERK-BL* mitogen extracellular-regulated-kinase phosphorylation blockade, *iPoCo* ischemic post-conditioning, *I/R* 30 min ischemia and 120 min reperfusion, *PI3K-BL* phosphatidylinositol(4,5)-bisphosphate-3-kinase blockade, *RISK-BL* reperfusion injury salvage kinase pathway blockade, *SAFE-BL* survival-activating factor enhancement pathway blockade, *T3* triiodothyronine at reperfusion, **a** T3 at reperfusion—given in increasing concentrations; *p < 0.001 vs. I/R; ^†^*p* < 0.01 vs. I/R + T3 (100 µg/L); one-way ANOVA with Fisher’s least significant differences post-hoc tests. **b** T3 at reperfusion—given under RISK or SAFE blockade; I/R and I/R + T3 (300 µg/L): groups are identical to those depicted in Fig. 1a; *p < 0.001 vs. I/R + T3 (300 µg/L); ^#^*p* < 0.01 vs. I/R + T3 (300 µg/L) + SAFE-BL; one-way ANOVA with Fisher’s least significant differences post-hoc tests

**Fig. 3:**
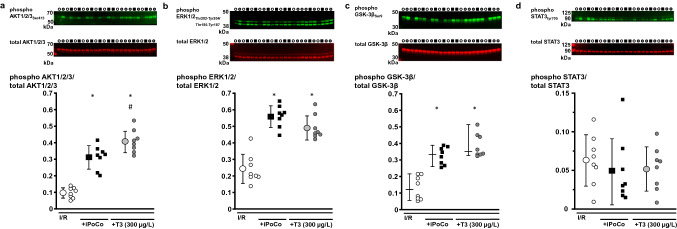
Phosphorylation of AKT1/2/3_Ser473_, ERK1/2_Thr202-Tyr204/Thr185-Tyr187_, GSK-3β_Ser9_ and STAT3_tyr705_ in isolated buffer-perfused rat hearts. Data are presented as means ± standard deviations. *iPoCo* ischemic post-conditioning, *I/R* ischemia and reperfusion, *T3* triiodothyronine at reperfusion. **a** Phosphorylation of protein kinase B (AKT) 1/2/3. Top; middle; bottom: fluorescence signal intensity of phosphorylated AKT1/2/3_Ser473_ (green); fluorescence signal intensity of total AKT1/2/3 (red); fluorescence signal intensity of phosphorylated protein was normalized to the respective total AKT1/2/3. *p < 0.01 vs. I/R; ^#^*p* < 0.01 vs. I/R + iPoCo; one-way ANOVA with Fisher’s least significant differences post-hoc tests. **b** Phosphorylation of mitogen extracellular-regulated-kinase (ERK) 1/2. Top; middle; bottom: fluorescence signal intensity of phosphorylated ERK1/2_Thr202**-**Tyr204/Thr185**-**Tyr187_ (green); fluorescence signal intensity of total ERK1/2 (red); fluorescence signal intensity of phosphorylated protein was normalized to the respective total ERK1/2. *p < 0.01 vs. I/R; one-way ANOVA with Fisher’s least significant differences post-hoc tests. **c** Phosphorylation of glycogen synthase kinase kinase (GSK)-3β. Data are presented as median (vertical line) with interquartile range (from 25 to 75%), horizontal line represents median. Top; middle; bottom: fluorescence signal intensity of phosphorylated GSK-3β_Ser9_ (green); fluorescence signal intensity of total GSK-3β (red); fluorescence signal intensity of phosphorylated protein was normalized to the respective total GSK-3β.*p < 0.05 vs. I/R; Kruskal–Wallis ANOVA on ranks with Tukey’s multiple comparisons procedures. **d** Phosphorylation of signal transducer and activator of transcription (STAT) 3 in myocardial biopsies. Top; middle; bottom: fluorescence signal intensity of phosphorylated STAT3_tyr705_ (green); fluorescence signal intensity of total STAT3 (red); fluorescence signal intensity of phosphorylated protein was normalized to the respective total STAT3

Baseline values for CF and LVDP were not different between groups (Online Resources, Online Tables 1 and 2). The recovery of CF at 10 min reperfusion was better with I/R + iPoCo than with I/R; whereas, I/R + T3 (100–500 µg/L) had no beneficial effect on CF. With I/R + T3 (500 µg/L), the recovery of LVDP during reperfusion was improved compared to I/R and similar to that with I/R + iPoCo. With I/R, infarct size was 36 ± 4% (Fig. 2a), and it was less with I/R + T3 (100 µg/L T3: 27 ± 15%; 200 µg/L T3: 24 ± 8%; 300 µg/L T3 14 ± 2%; 500 µg/L T3: 18 ± 6%, Fig. 2a). Infarct size reduction with I/R + T3 (300 and 500 µg/L) was similar to that with I/R + iPoCo (16 ± 7%, Fig. 2a). The myocardial levels of phosphorylated AKT1/2/3_Ser473_, ERK1/2_Thr202-Tyr204/Thr185-Tyr187_ and GSK-3β normalized to their respective total protein, were higher with I/R + T3 (300 µg/L) and with I/R + iPoCo, respectively, than with I/R (Fig. 3a–c). The levels of phosphorylated STAT3_Tyr705_, normalized to total STAT3 protein, were not different between groups (Fig. 3d).

In hearts subjected to I/R without T3 at reperfusion, RISK-BL, but not SAFE-BL, impaired the recovery of LVDP during reperfusion (Online Resources, Online Table 1). The blockers per se had no impact on infarct size (I/R + RISK-BL: 34 ± 9%; I/R + SAFE-BL: 32 ± 5%, Fig. 2b). Infarct size reduction by 300 µg/L T3 at reperfusion was abrogated by PI3K-BL, ERK-BL and RISK-BL, respectively (35 ± 12%; 29 ± 12%; 35 ± 10%, Fig. 2b). SAFE-BL had no impact on T3’s infarct size reduction (16 ± 5%, Fig. 2b).

### Viability of isolated cardiomyocytes

**Fig. 4:**
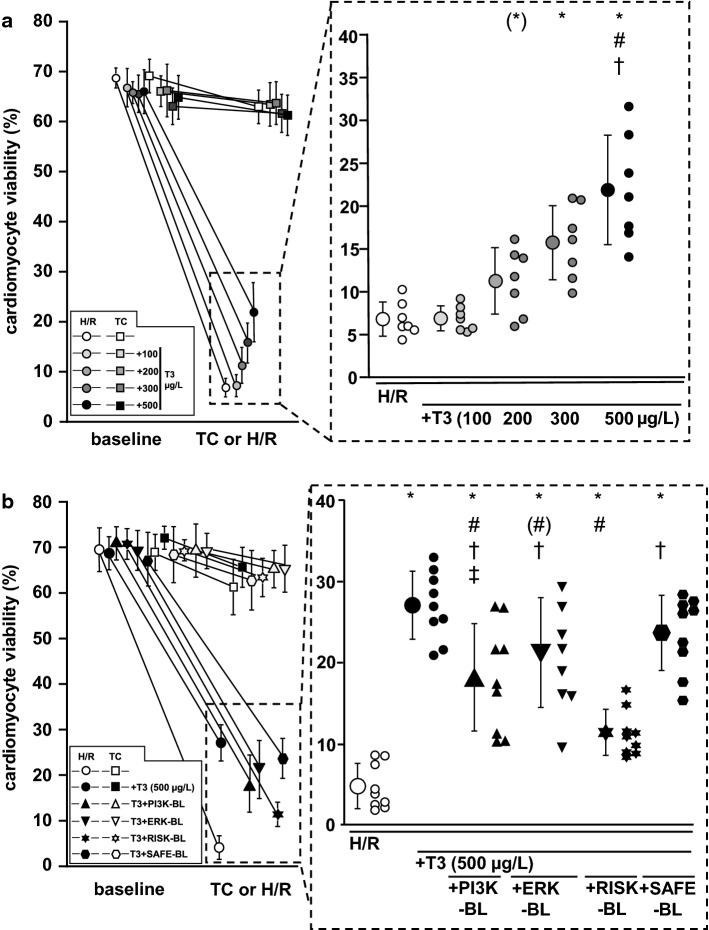
Impact of T3 at reoxygenation on cardiomyocytes viability. Data are presented as means ± standard deviations. *ERK-BL* mitogen extracellular-regulated-kinase blockade, *H/R* hypoxia/reoxygenation, *PI3K-BL* blockade of phosphatidylinositol(4,5)-bisphosphate-3-kinase, *RISK-BL* reperfusion injury salvage kinase pathway blockade, *SAFE-BL* signal transducer and activator of transcription pathway blockade, *T3* incubation with 100, 200, 300 or 500 µg/L triiodothyronine 5 min before and during reoxygenation, *TC* time control.** a** T3 at reoxygenation—incubation with increasing concentrations. Cardiomyocytes were isolated from *n* = 7 hearts. *p < 0.01 vs. H/R and H/R + T3 (100 µg/L); (*) *p* = 0.054 vs. H/R; ^#^*p* < 0.01 vs. H/R + T3 (200 µg/L); ^†^*p* = 0.01 vs. H/R + T3 (300 µg/L); one-way ANOVA for repeated measures with Fisher’s least significant differences post-hoc tests. **b** T3 at reoxygenation—incubation under PI3K-BL, ERK-BL, RISK-BL or SAFE-BL. Cardiomyocytes were isolated from *n* = 9 hearts. *p < 0.01 vs. H/R; ^#^*p* < 0.01 vs. H/R + T3 (500 µg/L); ^#^*p* = 0.015; ^†^*p* < 0.01 vs. H/R + T3 (500 µg/L) + RISK-BL; ^‡^*p* = 0.022 vs. H/R + T3 (500 µg/L) + SAFE-BL; one-way ANOVA for repeated measures with Fisher’s least significant differences post-hoc tests

The yield of viable cardiomyocytes was > 65% at baseline. After H/R, only 7 ± 2% of cardiomyocytes remained viable (Fig. 4a). The incubation with 100 µg/L T3 at reoxygenation had no impact on cardiomyocyte viability (7 ± 2%, Fig. 4a); whereas, the incubation with 200, 300 and 500 µg/L at reoxygenation preserved cardiomyocyte viability concentration-dependently (11 ± 4%; 15 ± 4%; 23 ± 6%, Fig. 4a). In TC experiments, the mean viability decreased by only 6 ± 2% (Fig. 4a). Incubation with T3 had no impact on cardiomyocyte viability in TC experiments, irrespectively of its concentration (Fig. 4a). Maximal protection of cardiomyocytes was induced by incubation with 500 µg/L T3 at reoxygenation. Therefore, 500 µg/L T3 was used in further experiments.

Also in blocker experiments, the yield of viable cardiomyocytes was > 65% at baseline. After H/R, only 5 ± 3% of cardiomyocytes remained viable (Fig. 4b). RISK-BL or SAFE-BL per se had no impact on cardiomyocyte viability after H/R (Online Resources, Online Fig. 2). PI3K-BL, ERK-BL and RISK-BL, respectively, attenuated the protection by 500 µg/L T3 (18 ± 6%; 21 ± 6%; 11 ± 3%, versus 27 ± 4%, Fig. 4b); whereas, SAFE-BL had no impact (24 ± 4%, Fig. 4b). In TC experiments, cardiomyocyte viability decreased by 8 ± 5%, irrespectively of the blockers (Online Resources, Online Fig. 2).

### Mitochondrial function

**Fig. 5:**
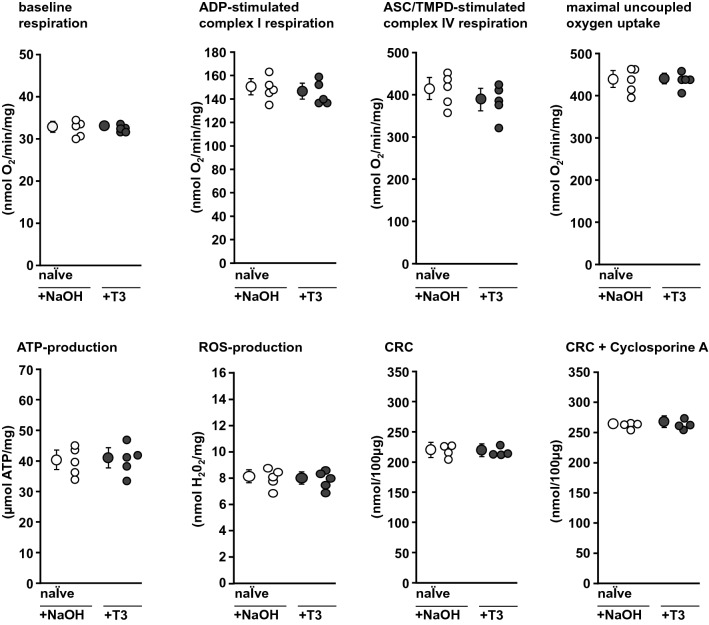
Functional parameters of mitochondria incubated with NaOH or T3, respectively, isolated from naïve rat hearts. Mitochondria were isolated from *n* = 5 hearts per group (for the respective measurements, see single data points). Data are presented as means ± standard deviations. *ADP* adenosine diphosphate, *ASC* ascorbate, *ATP* adenosine triphosphate, *CRC* calcium retention capacity, *NaOH* incubation with buffer and addition of NaOH, *ROS* reactive oxygen species, *T3* incubation with buffer and addition of 300 µg/L triiodothyronine, *TMPD* tetramethylphenylenediamine

**Fig. 6:**
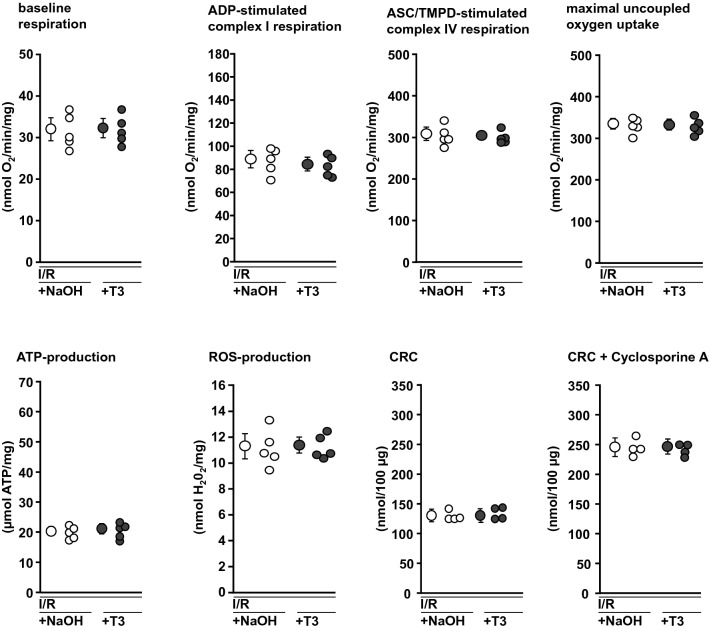
Functional parameters of mitochondria incubated with NaOH or T3, respectively, after isolation from buffer-perfused rat hearts subjected to 30 min ischemia and 10 min reperfusion. Mitochondria were isolated from *n* = 5 hearts per group (for the respective measurements, see single data points) hearts. Data are presented as means ± standard deviations. *ADP* adenosine diphosphate, *ASC* ascorbate, *ATP* adenosine triphosphate, *CRC* calcium retention capacity, *NaOH* incubation with buffer and addition of NaOH, *ROS* reactive oxygen species, *T3* incubation with buffer and addition of 300 µg/L triiodothyronine, *TMPD* tetramethylphenylenediamine

**Fig. 7:**
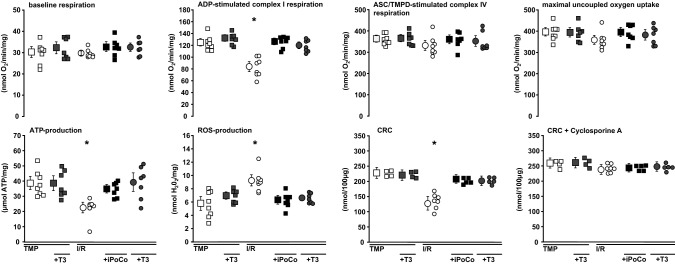
Functional parameters of mitochondria from buffer-perfused rat hearts subjected to I/R or TMP without or with iPoCo or T3, respectively, at reperfusion. Mitochondria were isolated from *n* = 7–8 hearts per group (for the respective measurements, see single data points). Data are presented as means ± standard deviations. *ADP* adenosine diphosphate, *ASC* ascorbate, *ATP* adenosine triphosphate, *CRC* calcium retention capacity, *iPoCo* ischemic post-conditioning, *I/R* 30 min ischemia and 120 min reperfusion with Krebs–Henseleit buffer, *ROS* reactive oxygen species, *T3* 300 µg/L triiodothyronine given at reperfusion or at the corresponding time point in TMP experiments, *TMP* time-matched perfusion without I/R, *TMPD* tetramethylphenylenediamine; *p < 0.01 vs. all other groups; one-way ANOVA with Fisher’s least significant differences post-hoc tests

Baseline respiration of mitochondria was not different between groups (Figs. 5, 6, 7). In mitochondria isolated from naïve hearts (Fig. 5) or buffer-perfused hearts subjected to I/R (Fig. 6) the in vitro incubation with T3 (300 µg/L) or NaOH, respectively, had no impact.

In mitochondria from hearts with TMP, 300 µg/L T3 had no effect. In mitochondria isolated after I/R, ADP-stimulated complex I respiration was decreased compared to mitochondria from hearts with TMP. With 300 µg/L T3 at reperfusion or iPoCo, respectively, this decrease in ADP-stimulated complex I respiration was reversed. Complex IV respiration and maximal uncoupled oxygen uptake were not different between groups, reflecting equal loading of viable mitochondria in the chamber. The decrease in ATP production by I/R was reversed with iPoCo or 300 µg/L T3, respectively, at reperfusion. Mitochondrial ROS formation was increased with I/R, and 300 µg/L T3 at reperfusion or iPoCo, respectively, attenuated such increase. The CRC impairment by I/R was reversed by 300 µg/L T3 or iPoCo, respectively, at reperfusion. With cyclosporine A, CRC was not different between groups (Fig. 7).

## Discussion

T3, when given at early reperfusion in a supraphysiological concentration, reduces infarct size in isolated buffer-perfused rat hearts through activation of the RISK, but not the SAFE pathway. The magnitude of cardioprotection and the involved signal transduction of T3’s cardioprotection are similar to those of iPoCo. T3’s cardioprotection is a cardiomyocyte phenomenon and associated with improved mitochondrial function.

We have chosen infarct size as the endpoint for cardioprotection [5], because it is a more robust endpoint than the improvement of LV function, and therefore extend the current knowledge on T3’s cardioprotection. In principle, we confirmed improved recovery of LV function with T3 at reperfusion. So far, no pathway has been identified as causal for cardioprotection by acute T3 treatment [4, 53]. Here, in the rat myocardium, T3’s cardioprotection causally involved the activation of AKT1/2/3 and ERK1/2 and inhibition of GSK-3β. Accordingly, infarct size reduction by T3 was attenuated by either PI3K-BL or ERK-BL, respectively, and abrogated when both blockers were combined—proving a causal role for the classical cardioprotective RISK pathway. We realize that RISK activation must occur during early reperfusion to induce protection; whereas, we have determined RISK activation by western blot after 120 min reperfusion along with infarct size; however, our blocker experiments covered the early reperfusion phase and thus provide evidence for a causal role of RISK activation in the observed protection by T3. SAFE pathway activation, however, was not involved in the cardioprotection by T3. Neither was STAT3 activated nor did SAFE-BL abrogate T3’s cardioprotection. In a prior study in isolated perfused rat hearts, T3 given at reperfusion decreased lactate dehydrogenase activity in the coronary effluent and reinforced mitophagy through a phosphatase tensin homolog-induced kinase 1/parkin-dependent mechanism, but no RISK activation was detected in myocardial biopsies taken after 45 min reperfusion using the western blot technique [4]. A higher dose of exogenous T3 in that study and differences in timing and processing of the myocardial biopsies [14] may explain this difference to our study. T3’s cardioprotection obviously utilizes the same pathways as iPoCo: in isolated perfused rat hearts iPoCo causally involves RISK pathway activation [66]. However, different post-conditioning strategies may involve different intracellular signaling pathways: i.e. pharmacological post-conditioning with sevoflurane [68], diazoxide [54], insulin [33], transforming growth factor-beta1 [2] and several other agents [21] also involves RISK pathway activation; whereas, hydrogen sulfide involves SAFE pathway activation [43] and oxytocin involves the activation of both pathways [58, 59] in isolated perfused rat hearts. Also, humoral transfer of ischemic conditioning’s cardioprotection in a post-conditioning mode involves SAFE, but not RISK pathway activation in isolated perfused rat hearts [41]. Thus, the combination of different cardioprotective strategies, e.g. the combination of pharmacological post-conditioning strategies to target both RISK and SAFE may enhance cardioprotection [9]. Of note, cardioprotective intracellular signaling is probably species-specific [9, 24, 36, 62]. In pigs with regional ischemia/reperfusion, iPoCo activates the SAFE pathway [35], and so far, no RISK activation by cardioprotective maneuvers has been demonstrated in pig myocardium [45, 62]. Furthermore, in the human myocardium cardioprotection by ischemic conditioning is associated with STAT5, but not with STAT3, AKT1/2/3 or ERK1/2 activation [31]. The NOS/protein kinase G pathway is known to be involved in cardioprotection [29]. Thus we cannot exclude that NOS is also involved in T3’s cardioprotection. Such hypothetical parallel activation of NOS appears less relevant in our study, since upstream RISK-BL abrogated T3’s cardioprotection completely.

To improve the transfer of promising preclinical cardioprotective strategies into clinical studies further characterization of possible species-specific differences in the signal transduction of pharmacological post-conditioning strategies is therefore mandatory.

We here used protein lysates of whole myocardium to characterize the RISK activation, which does not permit to differentiate between RISK activation in cardiomyocytes and other cell types such as vascular cells or fibroblasts. A prior experimental approach has indeed reported RISK activation by T3 in vascular cells [7]. Nevertheless, in our present study the involvement of RISK in T3’s cardioprotection was also demonstrated in cardiomyocytes and thus truly characterizes T3’s protection as a cardiomyocyte phenomenon. We therefore confirm prior results in neonatal cardiomyocytes [4], where cell death after H/R was also reduced by T3, although cardioprotection and its signaling are not comparable between adult cardiomyocytes and neonatal cardiomyocyte cell lines. In cultivated neonatal cardiomyocytes, the predominant pathomechanism of cell death is apoptosis; whereas, that in adult cardiomyocytes is necrosis [5]. The protective effect on cardiomyocytes may be paralleled by effects on vascular cells, rendering the coronary circulation another promising target for cardioprotection [20, 26]. In contrast to a prior study [4], we did not observe an improved recovery of CF in isolated buffer-perfused rat hearts with T3 treatment. Again, subtle differences in the experimental setting and higher T3 doses in the latter study [4] may account for this difference. In addition, the isolated buffer-perfused and denervated heart preparation with an artificially high CF and maximal vasodilation [42] may hamper the detection of T3’s protective effect on the coronary circulation.

Activation of RISK by cardioprotective post-conditioning strategies has been demonstrated to inhibit opening of the mPTP [10] through phosphorylation inhibition of GSK-3β and thus may link T3’s cardioprotective properties on the subcellular level to the preserved function of mitochondria. However, the role of GSK-3β for ischemic conditioning’s cardioprotection is not clear, since genetic ablation of GSK-3β did not abrogate cardioprotection by ischemic preconditioning in one study [49], but was mandatory for iPoCo’s cardioprotection in another study, which used the same animal model [17]. Of note, pharmacological inhibition of GSK-3β induces cardioprotection beyond mPTP inhibition [48].

Mitochondria also express T3 receptors, which may exert transcriptional effects [44]. However, exposure of isolated mitochondria neither from I/R injured buffer-perfused rat hearts nor from naïve rat hearts to T3 had an effect on mitochondrial function. Apparently, upstream activation of cardioprotective intracellular pathways is mandatory to transfer T3’s protection to the mitochondria.

In anecdotal reports of three patients treated for refractory hypothyroidism, when 1000 µg oral thyroxine was given on two consecutive days, no safety signals were observed within 3–6 days [37]. Also, in another case study of refractory hypothyroidism weekly intravenous 300 µg thyroxin over 14 months raised no safety signals [46]. These single human case studies are difficult to extrapolate to our saline-perfused isolated heart preparation, where no intestinal absorption and plasma-protein binding occurred [13]. Future studies in vivo and in clinically relevant large animal models are warranted to evaluate T3’s safety and efficacy before translation of T3’s adjunct cardioprotection to patients with myocardial infarction. Potentially then, in patients with acute myocardial infarction, a single intracoronary bolus application of T3 during PCI could be used to achieve a high local T3 concentration. Combination therapy of cardioprotective strategies has been proposed recently. In future studies, combined pharmacological and ischemic conditioning protocols such as the less invasive remote ischemic conditioning could serve as an attractive strategy to boost cardioprotection in patients with acute myocardial infarction [9].

## Supplementary Information

Below is the link to the electronic supplementary material.

Supplementary file1 (DOCX 32 KB)

Supplementary file2 (PPTX 9519 KB)

Supplementary file3 (PPTX 39 KB)

Supplementary file4 (DOCX 24 KB)

## Data Availability

All data are available from the corresponding author upon reasonable request. Unedited western blot data are included in the electronic supplementary material.
