# Supplementary material for: Cardioprotection by post-conditioning with exogenous triiodothyronine in isolated perfused rat hearts and isolated adult rat cardiomyocytes
Source: Basic Res Cardiol. 2021 Apr 19;116(1):27. doi: 10.1007/s00395-021-00868-6 (PMC8055637; doi:10.1007/s00395-021-00868-6)
Supplement: Supplementary file 1 — Supplementary file1 (DOCX 32 KB) [file 395_2021_868_MOESM1_ESM.docx]

**SUPPLEMENTARY MATERIAL**

**Cardioprotection by post-conditioning with exogenous triiodothyronine in isolated perfused rat hearts and isolated adult rat cardiomyocytes**

Helmut Raphael Lieder, Felix Braczko, Nilgün Gedik, Merlin Stroetges, Gerd Heusch, Petra Kleinbongard

Institute for Pathophysiology, West German Heart and Vascular Center, University of Essen Medical School, Essen, Germany

**Methods**

**Isolated buffer-perfused heart**

Male Lewis rats (200-380 g, 2.0-3.5 months, central Animal Laboratory, University of Duisburg-Essen, Medical School, Essen, Germany) were sacrificed by intraperitoneal injection of sodium pentobarbital (800 mg/kg, narcoderm, CP-Pharma, Burgdorf, Germany). Hearts were excised, arrested in cold saline (8°C, supplemented with unfractionated heparin 300 IU/Ml) and weighed. The aorta was immediately cannulated, and hearts were mounted on a Langendorff-apparatus and perfused with modified Krebs-Henseleit buffer (in mmol/L: NaCl 118.0, KCl 4.7, MgSO_4_ 16.0, KH_2_PO_4_ 1.2, glucose 5.6, NaHCO_3_ 24.9, sodium pyruvate 2.0, CaCl_2_ 2.0; gassed with 95% O_2_ and 5% CO_2_ in a pre-warmed reservoir, pH 7.40) at constant pressure of 65-70 mmHg. Coronary flow (CF) was measured with an inline ultrasonic flowprobe (TS410, Transsonic Systems Inc., Ithaca, NY, USA) above the aortic cannula. A fluid-filled latex balloon was inserted into the left ventricular (LV) cavity and connected to a pressure transducer (Codan-PVB, Lensahn, Germany) to measure LV pressure. End-diastolic LV pressure was set to 5-15 mmHg at baseline by graded balloon inflation during the initial 5 min perfusion. LV developed pressure (LVDP) was calculated as the difference between peak and end-diastolic LV pressure. CF, end-diastolic and peak LV pressure were continuously recorded (LabChart 8, AD Instruments Pty LTD, New South Wales, Australia). Hearts were allowed to stabilize for 10-20 min. Preparations with CF <10.0 mL/min or >18.0 min or LVDP <60 mmHg after the stabilization period were excluded[38-41]. Twelve isolated heart preparations in total did not meet baseline criteria and were thus excluded from further analysis. Heart rate was kept constant at 360 beats per min by right atrial pacing. Hearts were immersed in pre-warmed oxygenated Krebs-Henseleit buffer. The temperatures of the perfusion and immersion buffers were monitored with probes in the aortic cannula and in the immersion buffer chamber throughout the experiment and kept between 37.5°C and 37.8°C by heat exchangers. Ischemia and reperfusion (I/R) was induced by 30 min full stop of perfusion and subsequent 120 min reperfusion. CF and LVDP were calculated as mean values during the last min each of the stabilization period (baseline), at 5 and 25 min ischemia and at 10, 30 and 60 min reperfusion, respectively. After completion of reperfusion, the apex of each heart was cut off (~50-100 mg) and quickly frozen in liquid nitrogen for later protein analysis by western blot. The hearts were frozen in Cryomatrix^TM^ (Thermo Fisher Sientific, Schwerte, Germany) at -20°C and cut into transverse 2 mm thick slices. Infarcted tissue was demarcated by staining with 0.09 mol/L sodium phosphate buffer containing 1.5% triphenyl tetrazolium chloride (TTC) at 37°C for 5 min. Stained slices were photographed from both sides. The total slice area and the infarcted areas were quantified by computer-assisted planimetry (ImageJ 1.48v, National Institutes of Health, Bethesda, Maryland, USA), and infarct size was calculated as percent of the sum of left and right ventricular mass (% of ventricular mass). The experimental groups and protocols in isolated buffer-perfused hearts are summarized in Fig. 1 a.

**Phosphorylation of cardioprotective proteins**

Protein phosphorylation was analyzed in biopsies taken from the isolated buffer-perfused hearts after 120 min reperfusion. From hearts with I/R, I/R+ischemic post-conditioning (iPoCo) or I/R+triiodothyronine (T3, 300 µg/L), respectively, n=8 myocardial tissue biopsies were randomly selected to have them all analyzed on the same gel/membrane. Myocardial tissue biopsies (~2-10 mg) were homogenized in 100.0 mmol/L tris(hydroxymethyl)aminomethane with 2% sodium dodecyl sulfate, (w/v; SERVA Electrophoresis GmbH, Heidelberg, Germany), heated to 70°C for 5 min and centrifuged at 16.000 xg for 10 min. The protein lysate containing supernatants were stored at -80°C in aliquots. In preliminary experiments, for each analyzed protein and its phosphorylated form, the combined linear range had been determined according to the manufacturers protocol[55], using the dilution series of: 30, 15, 7.5, 3.75, 1.98, 0.94 and 0.47 µg of protein lysate. The determined protein quantity within the linear range was then used for western blotting. Protein lysate aliquots of 13 µg for protein kinase B (AKT), 20 µg for mitogen extracellular-regulated-kinase 1/2 (ERK1/2), 22 µg for glycogen synthase kinase (GSK)-3β and 20 µg for signal transducer and activator of transcription (STAT3) were electrophoretically separated on precast stain-free 12% (for AKT1/2/3, ERK1/2 and STAT3) or 7.5% (for GSK-3β) sodium dodecyl sulfate polyacrylamide electrophoresis gels (BioRad, Hercules, USA).

Total protein was visualized by an ultraviolet light-induced fluorescence reaction of protein-tryptophan with tri-halocompounds within the stain-free gels and imaged using the Gel Doc EZ system (BioRad). Proteins were transferred to 0.45 µm low fluorescence polyvinylidene difluoride membranes (Merck, Chemicals GmbH, Darmstadt, Germany) using the Trans-Blot Turbo™ transfer system (BioRad). The membranes were imaged (Gel Doc EZ system, BioRad) and cut horizontally into three parts (Online Resources, Online Fig. 1). After reactivation with 100% methanol and blocking (Intercept® blocking buffer, LI-COR Biosciences, Lincoln, USA) for one hour at room temperature, membrane fragments were rinsed with tris-buffered saline. Then membranes were incubated with the respective primary antibodies directed against the phosphorylated forms of AKT1/2/3_Ser473_ (Cell Signaling, Danvers, USA #9271, 1:150), ERK1/2_Thr202-Tyr204/Thr185-Tyr187_ (Cell Signaling, #9101, 1:500), GSK-3β_Ser9_ (Cell Signaling, #9336, 1:250) or STAT3_Tyr705_ (Cell Signaling, #9138, 1:250) over night at 4°C. Membranes were washed 4x for 5 min with tris-buffered saline containing polyoxyethylene-20-sorbitan monolaurate (TBST) before being incubated with antibodies directed against the total forms of AKT1/2/3 (Cell Signaling, #5829, 1:1000), ERK1/2 (Cell Signaling, #4696, 1:500), GSK-3β (BD Biosciences, Franklin Lakes, USA, #610202, 1:500) or STAT3 (Cell signaling, #9139, 1:500) for one hour at room temperature. Membranes were again washed for 4x 5 min with TBST before one hour incubation with the secondary antibodies (LI-COR Biosciences: IRDye® 800CW goat anti-mouse IgG1, 1:5000, for phosphorylated STAT3; IRDye® 680LT goat anti-mouse IgG2a, 1:20000, for total STAT3; IRDye® 800CW goat anti-rabbit IgG, 1:5000 for phosphorylated AKT1/2/3 and phosphorylated GSK-3β, 1:20000 for phosphorylated ERK1/2; IRDye® 680RD goat anti-mouse IgG, 1:20000 for total AKT1/2/3 and total ERK1/2, 1:5000 for total GSK-3β). Primary antibodies were diluted in TBST containing 5% bovine serum albumin. Secondary antibodies were diluted in Intercept® blocking buffer (LI-COR Biosciences) supplemented with 0.2% polyoxyethylene-20-sorbitan monolaurate.

Fluorescence signal intensity of phosphorylated proteins and their respective total proteins was imaged using the LI-COR Biosciences infrared imaging system. Detected signals were analyzed with the LI-COR Biosciences Empiria® studio software (version 1.3.0.83). The fluorescence signal intensity signal of each phosphorylated protein was normalized to that of the respective total protein. After fluorescence signal intensity detection, each membrane fragment was stained with Ponceau S as an additional loading/transfer control (Online Resources, Online Fig.1).

**Isolated adult ventricular cardiomyocytes**

Male Lewis rats (200-380 g, 2.0-3.5 months, central Animal Laboratory, University of Duisburg-Essen, Medical School, Essen, Germany) were sacrificed, and hearts isolated, as described above. Hearts were perfused with Tyrode solution (in mmol/L: 113.0 NaCl, 4.7 KCl, 0.6 KH_2_PO_4_, 0.6, Na_2_HPO4, 1.2 MgSO_4_, 12.0 NaHCO_3_, 10.0 KHCO_3_, 10.0 4-(2-hydroxyethyl)-1-piperazine ethanesulfonic acid, 30.0 taurine, 5.5 glucose, and 10.0 2,3-butanedione monoxime, pH 7.42 at 36.5 °C) at constant flow (7 mL/min x mg heart weight) for 3 min. Liberase (Liberase TM Research Grade, Roche, Basel, Swiss Confederation) and 12.5 µmol/L CaCl_2_ were subsequently added to the perfusion buffer, and hearts were digested for 4 min 30 s. Atrial and connective tissue was removed and discarded, ventricles were sectioned, and cells were re-suspended in Tyrode solution containing 10% bovine calf serum and 12.5 µmol/L CaCl_2_. Cardiomyocytes were isolated, separated from tissue residues by filtering through a nylon mesh filter (200 µm pore size, Millipore, Billerica, USA), and CaCl_2_ was slowly titrated at 20°C to a final concentration of 1 mmol/L (5 steps each of 10 min duration). Cardiomyocytes were kept in normoxic buffer (containing in mmol/L: 125.0 NaCl, 5.4 KCl, 1.2 NaH_2_PO_4_, 20.0 4-(2-hydroxyethyl)-1-piperazine ethanesulfonic acid, 5.0 taurine, 15.0 glucose, 2.5 creatine, 0.5 MgCl_2_, and 1.0 CaCl_2_, gassed with 100% oxygen, pH 7.4) for 5 min before viability was determined at baseline. In experimental set 1 (Fig. 1 b), cardiomyocytes from each heart were divided into hypoxia/reoxygenation (H/R) and time control (TC) groups. Hypoxia was induced for 30 min by exposing cardiomyocytes to glucose-free hypoxic buffer (in mmol/L: 119.0 NaCl, 120.0 KCl, 5.0 4-(2-hydroxyethyl)-1-piperazine ethanesulfonic acid, 0.5 MgCl_2_, 0.9 CaCl_2_, 20.0 sodium lactate, pH 6.5) and sealing with mineral oil; cells were kept in solution where they sediment; reoxygenation was induced by removal of oil and hypoxic buffer and by adding reoxygenation buffer with an osmolality of 250 mosm/L (in mmol/L: 88.0 NaCl, 5.4 KCl, 1.2 NaH_2_PO4, 12.0 NaHCO_3_, 20.0 4-(2-hydroxyethyl)-1-piperazine ethanesulfonic acid, 5.0 taurine, 15.0 glucose, 2.5 creatine, 0.5 MgCl_2_, and 1.0 CaCl_2_, gassed with 100% oxygen, pH 7.4) for 5 min. In TC experiments, cardiomyocytes were exposed to normoxic buffer for 65 min. Cardiomyocyte samples were taken at 5 min (baseline) and again at 65 min after H/R and TC in all experimental groups, respectively. The impact of T3 on cardiomyocyte viability in H/R groups was studied by adding T3 (100, 200, 300, 500 µg/L) to the hypoxia buffer at 25 min hypoxia and to the reoxygenation bufferand in TC groups to normoxic buffers, respectively. In experimental set 2 (Fig. 1 b), cardiomyocytes were isolated and divided into H/R and TC groups without or with 500 µg/L T3. In subgroups of H/R and TC, experiments were performed under pharmacological blockade of the reperfusion injury salvage kinase (RISK) pathway or the survival activating factor enhancement pathway (SAFE) by adding blockers to all buffers throughout the entire experiment. To further distinguish between the kinases of the RISK pathway, the phosphatidylinositol(4,5)-bisphosphate-3-kinase **(**PI3K) blocker wortmannin or the ERK1/2 blocker U0126 were also added separately to the buffers.

Cardiomyocytes were stained with 0.5% trypan blue, and 400-800 cells per sample were analyzed in non-overlapping visual fields using light microscopy at 40 x magnification (Leica DMLB microscope, Leica, Bensheim, Germany). Viability was expressed as the percentage of rod-shaped, unstained cardiomyocytes over the total number of cells. Cardiomyocyte preparations with a viability of <60% at baseline were discarded (n = 3 preparations). When viability in TC experiments decreased by >15%, data of this respective preparation were excluded from the analyses (n=5 preparations).

**Isolated mitochondria**

In experimental set 1, mitochondria were isolated form naïve hearts or from buffer-perfused hearts with I/R. Hearts were arrested in ice-cold isolation buffer. Isolation buffer contained (in mmol/L): 250.0 sucrose, 10.0 (4-(2-hydroxyethyl)-1-piperazine ethanesulfonic acid, 1.0 ethylene glycol tetraacetic acid, pH 7.4, supplemented with 0.5% (w/v) bovine serum albumin and proteinase inhibitors (cOmplete™, Roche) and phosphatase inhibitors (PhosSTOP™, Roche). Hearts were minced thoroughly using scissors and then homogenized with a tissue homogenizer (Ultra‐Turrax, IKA, Staufen, Germany) using two 10 s treatments at a shaft rotation rate of 6.500 rpm to release subsarcolemmal mitochondria. Tissue processing was performed at 4°C. The tissue was further homogenized with a Teflon pestle to release interfibrillar mitochondria. The homogenate containing subsarcolemmal and interfibrillar mitochondria was centrifuged at 700 xg for 10 min. The supernatant was collected and centrifuged at 14.000 xg for 10 min. The resulting pellet was resuspended in isolation buffer without bovine serum albumin and centrifuged at 10.000 xg for 5 min. The last procedure was repeated, and the pellet was divided and resuspended in 700 μL of isolation buffer. Mitochondria were incubated with 4 µmol/L NaOH or with 300 µg/L T3, respectively, for 10 min before functional measurements were performed. The protein concentration of the resuspended pellets was determined using a protein assay (Lowry method; Bio‐Rad, Hercules, USA) with bovine serum albumin as standard (Thermo Scientific, Waltham, USA). In experimental set 2, the experimental protocols as in groups I/R, I/R+iPoCo, and I/R+T3 (300 µg/L), respectively, were repeated, and reperfusion was stopped after 10 min (Fig. 1 c). For the respective control experiments on mitochondrial function, hearts were perfused for 30 min, followed either by 10 min perfusion without (time-matched perfusion, TMP) or with 300 µg/L T3 (TMP+T3). Mitochondria were isolated at 10 min reperfusion or at the corresponding time point in TMP experiments, respectively.

***Mitochondrial respiration***

Oxygen consumption was measured with a Clark‐type electrode (Strathkelvin, Glasgow, UK) at 37°C during magnetic stirring in incubation buffer (in mmol/L: 125.0 KCl, 10.0 3-(N-morpholino)propanesulfonic acid, 2.0 MgCl_2_, 5.0 KH_2_PO_4_, and 0.2 ethylene glycol tetraacetic acid, with 5.0 glutamate and 5.0 malate as substrates for complex I). The electrode was calibrated using the solubility coefficient of 217 µmol/L O_2_ at 37°C. For the measurement of complex I respiration, suspended mitochondria (corresponding to a protein mass of 50 μg) were added to 0.5 mL of incubation buffer. After 2 min, adenosine diphosphate (ADP, 0.4 mmol/L) was added, and ADP‐stimulated respiration was measured over 2 to 3 min. Complex IV respiration was stimulated by adding N,N,N,N’-tetramethyl-p-phenylenediamine (TMPD, 300 μmol/L) and ascorbate (3 mmol/L), which donates electrons to cytochrome oxidase through reduction of cytochrome c. Maximal uncoupled oxygen uptake was measured in the presence of 30 nmol/L of carbonyl cyanide-p-trifluoro-methoxyphenyl-hydrazone (FCCP).

***Mitochondrial ATP production***

Suspended mitochondria (corresponding to a protein mass of 50 μg) were added to 0.5 mL of incubation buffer in a tube on a heat block at 37°C. After 2 min, ADP (0.4 mmol/L) was added and incubated for 3 min. The incubation buffer containing mitochondria was taken from the tube and immediately supplemented with ATP assay mix (diluted 1:5). Mitochondrial ATP production was determined immediately and compared with ATP standards using a 96‐well white plate and a spectrophotometer (F-7100, Hitachi High-Tech, Krefeld, Germany) at 560 nm emission wavelength.

***Mitochondrial reactive oxygen species (ROS) formation***

The amplex™ red hydrogen peroxide assay (Thermo Fisher Scientific, Waltham, USA) was used to determine ROS concentration in the extramitochondrial space. Amplex™ red reacts in a 1:1 stoichiometry with peroxides under catalysis by horseradish peroxidase and produces highly fluorescent resorufin. Suspended mitochondria (corresponding to a protein mass of 50 μg) were added to 0.5 mL of incubation buffer in a tube on a heat block at 37°C. After 2 min, ADP (0.4 mmol/L) was added and incubated for 3 min. The incubation buffer containing mitochondria and ADP was supplemented with 50 μmol/L of amplex™ red and 2 U/mL of horseradish peroxidase. The supernatant was collected after 120 min incubation in the dark. ROS concentration was determined and compared with H_2_O_2_ standards using a 96‐well black plate and a fluorescence spectrophotometer (F-7100, Hitachi High-Tech, Krefeld, Germany) at excitation and emission wavelengths of 490 and 580 nm, respectively.

***Calcium retention capacity (CRC)***

CRC was determined using suspended mitochondria (corresponding to a protein mass of 100 µg) in 1 mL buffer (in mmol/L: 125.0 KCl, 10.0 3-(N-morpholino)propanesulfonic acid, 2.0 MgCl_2_, 5.0 KH_2_PO_4_ with 5.0 glutamate and 5.0 malate as substrates in the presence of ADP (0.4 mmol/L) at 37°C). Calcium green™-5N (0.5 µmol/L, Thermo Fisher Scientific) was used to measure the extramitochondrial calcium concentration in a spectrophometer (Cary Eclipse, Varian, Mulgrave, Victoria, Australia) at 500 nm emission and 530 nm extinction wavelengths, respectively. Pulses of 5 nmol/L CaCl_2_ were added (1/min) until a rapid increase in calcium green fluorescence indicated mitochondrial permeability transition pore (mPTP) opening[15]. Cyclosporine A delays mPTP opening by interaction with cyclophilin D to keep the pore closed. Therefore, additional measurements in the presence of cyclosporine A (10 µmol/L) served as control[1, 15].

**References**

1. Basso E, Fante L, Fowlkes J, Petronilli V, Forte MA, Bernardi P (2005) Properties of the permeability transition pore in mitochondria devoid of cyclophilin d. J Biol Chem 280: 18558-18561 doi:10.1074/jbc.C500089200

15. Gedik N, Maciel L, Schulte C, Skyschally A, Heusch G, Kleinbongard P (2016) Cardiomyocyte mitochondria as targets of humoral factors released by remote ischemic preconditioning. Arch Med Sci 13: 448-458 doi:doi: 10.5114/aoms.2016.61789

38. Lieder HR, Baars T, Kahlert P, Kleinbongard P (2016) Aspirate from human stented saphenous vein grafts induces epicardial coronary vasoconstriction and impairs perfusion and left ventricular function in rat bioassay hearts with pharmacologically induced endothelial dysfunction. Physiol Rep 4: e12874 doi:10.14814/phy2.12874

39. Lieder HR, Irmert A, Kamler M, Heusch G, Kleinbongard P (2019) Sex is no determinant of cardioprotection by ischemic preconditioning in rats, but ischemic/reperfused tissue mass is for remote ischemic preconditioning. Physiol Rep 7: e14146 doi:10.14814/phy2.14146

40. Lieder HR, Kleinbongard P, Skyschally A, Hagelschuer H, Chilian WM, Heusch G (2018) Vago-splenic axis in signal transduction of remote ischemic preconditioning in pigs and rats. Circ Res 123: 1152-1163 doi:10.1161/CIRCRESAHA.118.313859

41. Lieder HR, Skyschally A, Heusch G, Kleinbongard P (2019) Plasma from remotely conditioned pigs reduces infarct size when given before or after ischemia to isolated perfused rat hearts. Pflügers Arch 471: 1371-1379 doi:10.1007/s00424-019-02314-y

55. Pillai-Kastoori L, Schutz-Geschwender AR, Harford JA (2020) A systematic approach to quantitative western blot analysis. Anal Biochem 593: 113608 doi:10.1016/j.ab.2020.113608
