# Supplementary material for: Cardioprotection by post-conditioning with exogenous triiodothyronine in isolated perfused rat hearts and isolated adult rat cardiomyocytes
Source: Basic Res Cardiol. 2021 Apr 19;116(1):27. doi: 10.1007/s00395-021-00868-6 (PMC8055637; doi:10.1007/s00395-021-00868-6)
Supplement: Supplementary file 2 — Supplementary file2 (PPTX 9519 KB) [file 395_2021_868_MOESM2_ESM.pptx]

## Slide 1
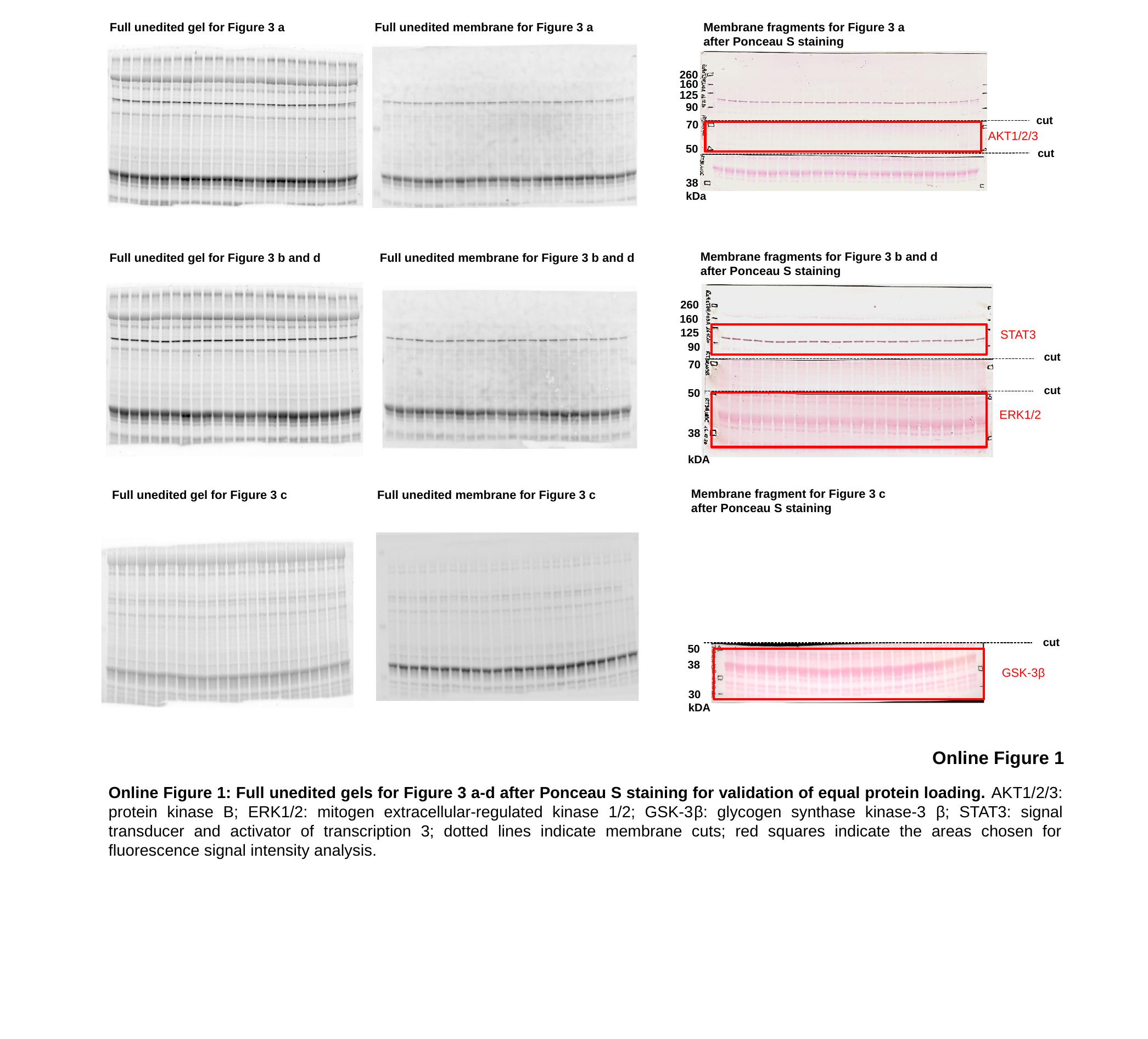

Full unedited membrane for Figure 3 a
Membrane fragments for Figure 3 a
after Ponceau S staining
260
160
125
90
cut
70
AKT1/2/3
50
cut
38
kDa
Full unedited gel for Figure 3 a
Membrane fragments for Figure 3 b and d
after Ponceau S staining
Full unedited gel for Figure 3 b and d
Full unedited membrane for Figure 3 b and d
260
125
90
70
50
38
kDA
160
STAT3
cut
cut
ERK1/2
Online Figure 1
Online Figure 1: Full unedited gels for Figure 3 a-d after Ponceau S staining for validation of equal protein loading. AKT1/2/3: protein kinase B; ERK1/2: mitogen extracellular-regulated kinase 1/2; GSK-3β: glycogen synthase kinase-3 β; STAT3: signal transducer and activator of transcription 3; dotted lines indicate membrane cuts; red squares indicate the areas chosen for fluorescence signal intensity analysis.
Membrane fragment for Figure 3 c
after Ponceau S staining
Full unedited gel for Figure 3 c
Full unedited membrane for Figure 3 c
cut
50
38
30
kDA
GSK-3β
