# Supplementary figures and images for: Cardioprotection by post-conditioning with exogenous triiodothyronine in isolated perfused rat hearts and isolated adult rat cardiomyocytes
Source: Basic Res Cardiol. 2021 Apr 19;116(1):27. doi: 10.1007/s00395-021-00868-6 (PMC8055637; doi:10.1007/s00395-021-00868-6)

## Slide 1
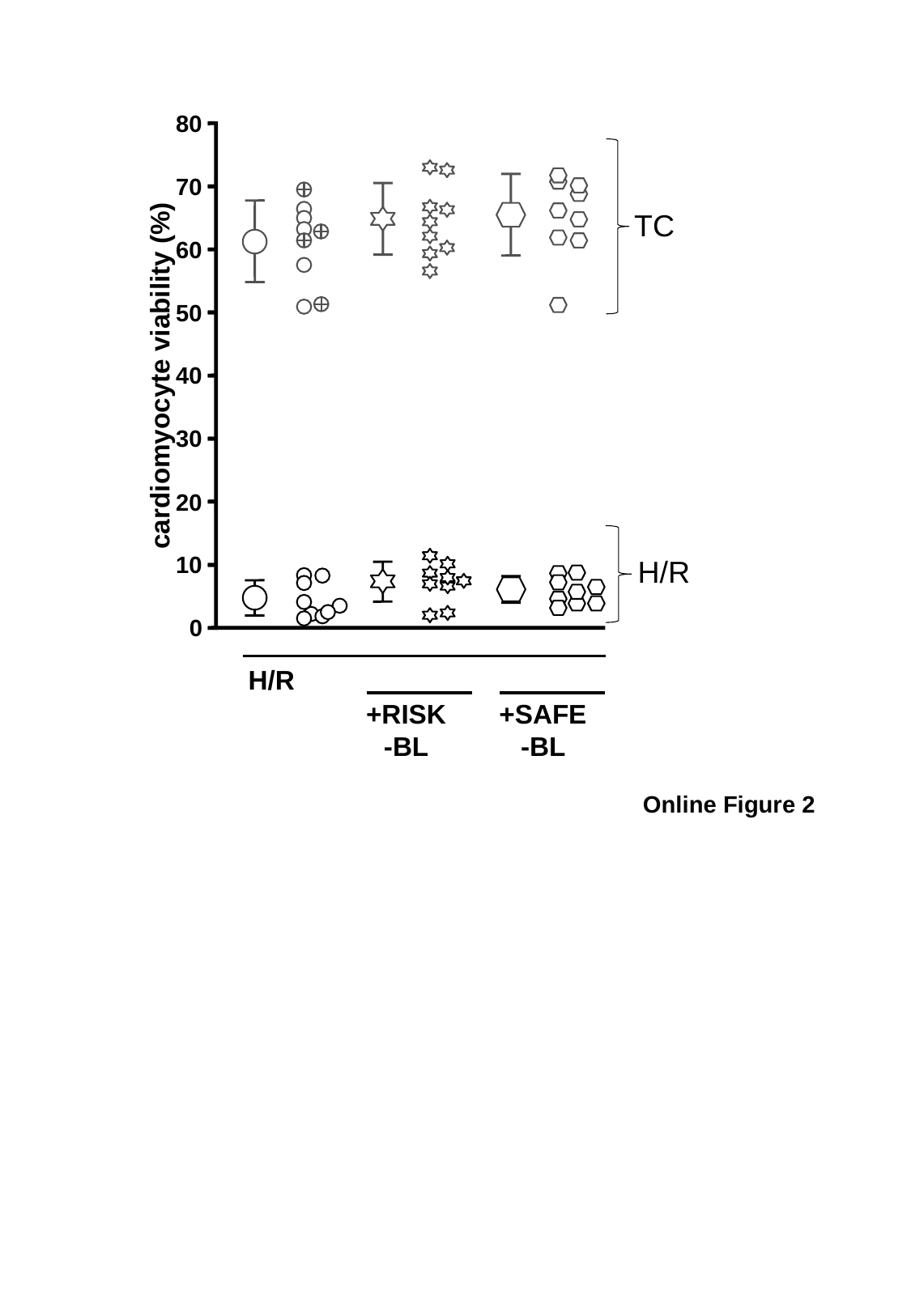

80
70
60
50
40
30
20
10
TC
cardiomyocyte viability (%)
H/R
0
H/R
+RISK
-BL
+SAFE
-BL
Online Figure 2

Supplement: Supplementary file 3 — Supplementary file3 (PPTX 39 KB) [file 395_2021_868_MOESM3_ESM.pptx]
