# Supplementary material for: Cardioprotection by post-conditioning with exogenous triiodothyronine in isolated perfused rat hearts and isolated adult rat cardiomyocytes
Source: Basic Res Cardiol. 2021 Apr 19;116(1):27. doi: 10.1007/s00395-021-00868-6 (PMC8055637; doi:10.1007/s00395-021-00868-6)
Supplement: Supplementary file 4 — Supplementary file4 (DOCX 24 KB) [file 395_2021_868_MOESM4_ESM.docx]

**Online Table 1: CF and LVDP of isolated buffer-perfused rat hearts**

| Groups for infarct size measurement | Time | CF  (ml/min) | | | LVDP (mmHg) | | |
| --- | --- | --- | --- | --- | --- | --- | --- |
| I/R (n=10) | baseline | 14.4 | ± | 1.7 | 102 | ± | 16 |
|  | isch5 | 0.0 | ± | 0.0* | 1 | ± | 1* |
|  | isch25 | 0.0 | ± | 0.0* | 2 | ± | 3* |
|  | rep10 | 9.0 | ± | 2.9*^(†)^ | 24 | ± | 17*^§^ |
|  | rep30 | 9.5 | ± | 2.9*^†^ | 36 | ± | 15*^†§^ |
|  | rep60 | 9.7 | ± | 2.7* | 35 | ± | 12*^§^ |
| I/R+iPoCO | baseline | 14.3 | ± | 1.9 | 106 | ± | 17 |
| (n=15) | isch5 | 0.0 | ± | 0.0* | 2 | ± | 5* |
|  | isch25 | 0.0 | ± | 0.0* | 6 | ± | 8* |
|  | rep10 | 10.9 | ± | 2.7* | 38 | ± | 22*^‡^ |
|  | rep30 | 11.3 | ± | 2.6* | 48 | ± | 25*^†^ |
|  | rep60 | 10.5 | ± | 1.8* | 55 | ± | 9*^†^ |
| I/R+T3 (100 µg/L) | baseline | 14.7 | ± | 1.9 | 115 | ± | 11 |
| (n=12) | isch5 | 0.0 | ± | 0.0* | 1 | ± | 1* |
|  | isch25 | 0.0 | ± | 0.0* | 2 | ± | 3* |
|  | rep10 | 7.7 | ± | 1.6*^‡^ | 16 | ± | 13*^§^ |
|  | rep30 | 8.5 | ± | 1.5*^†^ | 18 | ± | 16*^§^ |
|  | rep60 | 8.3 | ± | 1.2*^†^ | 23 | ± | 15*^§^ |
| I/R+T3 (200 µg/L) | baseline | 13.8 | ± | 1.3 | 106 | ± | 9 |
| (n=13) | isch5 | 0.0 | ± | 0.0* | 1 | ± | 1* |
|  | isch25 | 0.0 | ± | 0.0* | 2 | ± | 2* |
|  | rep10 | 9.1 | ± | 2.3*^‡^ | 23 | ± | 18*^§^ |
|  | rep30 | 9.5 | ± | 2.2* | 41 | ± | 21*^‡^ |
|  | rep60 | 9.5 | ± | 2.0*^†^ | 39 | ± | 19*^†^ |
| I/R+T3 (300 µg/L) | baseline | 13.7 | ± | 1.5 | 108 | ± | 18 |
| (n=12) | isch5 | 0.0 | ± | 0.0* | 1 | ± | 0* |
|  | isch25 | 0.0 | ± | 0.0* | 1 | ± | 3* |
|  | rep10 | 8.8 | ± | 1.5*^‡^ | 25 | ± | 21*^§^ |
|  | rep30 | 9.3 | ± | 1.3*^‡^ | 37 | ± | 15*^‡§^ |
|  | rep60 | 9.5 | ± | 1.4*^‡^ | 41 | ± | 11*^†^ |
| I/R+T3 (500 µg/L) | baseline | 14.3 | ± | 2.6 | 105 | ± | 13 |
| (n=11) | isch5 | 0.0 | ± | 0.0* | 1 | ± | 1* |
|  | isch25 | 0.0 | ± | 0.0* | 1 | ± | 2* |
|  | rep10 | 9.5 | ± | 1.9*^‡^ | 41 | ± | 21* |
|  | rep30 | 9.4 | ± | 2.5* | 49 | ± | 21* |
|  | rep60 | 9.3 | ± | 1.9* | 50 | ± | 15* |
| I/R+RISK-BL | baseline | 15.0 | ± | 2.0 | 104 | ± | 23 |
| (n=9) | isch5 | 0.0 | ± | 0.0* | 1 | ± | 1* |
|  | isch25 | 0.0 | ± | 0.0* | 1 | ± | 1* |
|  | rep10 | 9.2 | ± | 2.1* | 17 | ± | 18*^§^ |
|  | rep30 | 9.2 | ± | 1.8* | 18 | ± | 17*^#†‡§^ |
|  | rep60 | 9.1 | ± | 1.5* | 19 | ± | 16*^#†‡§^ |
| I/R+SAFE-BL | baseline | 14.9 | ± | 1.7 | 111 | ± | 13 |
| (n=10) | isch5 | 0.0 | ± | 0.0* | 1 | ± | 0* |
|  | isch25 | 0.0 | ± | 0.0* | 3 | ± | 6* |
|  | rep10 | 9.3 | ± | 2.3* | 28 | ± | 22* |
|  | rep30 | 10.2 | ± | 2.2* | 38 | ± | 24* |
|  | rep60 | 9.7 | ± | 1.7* | 44 | ± | 14* |
| I/R+T3 (300 µg/L)+PI3K-BL | baseline | 14.7 | ± | 2.4 | 102 | ± | 17 |
| (n=10) | isch5 | 0.0 | ± | 0.0* | 1 | ± | 0* |
|  | isch25 | 0.0 | ± | 0.0* | 2 | ± | 2* |
|  | rep10 | 9.4 | ± | 1.6* | 13 | ± | 15*^†‡§^ |
|  | rep30 | 9.3 | ± | 1.6* | 21 | ± | 17*^#†‡§^ |
|  | rep60 | 9.5 | ± | 2.2* | 26 | ± | 16*^†‡§^ |
| I/R+T3 (300 µg/L)+ERK-BL | baseline | 14.4 | ± | 1.6 | 109 | ± | 16 |
| (n=10) | isch5 | 0.0 | ± | 0.0* | 1 | ± | 1* |
|  | isch25 | 0.0 | ± | 0.0* | 4 | ± | 4* |
|  | rep10 | 7.7 | ± | 2.4* | 14 | ± | 14*^‡§^ |
|  | rep30 | 8.7 | ± | 2.7* | 21 | ± | 21*^#†‡§^ |
|  | rep60 | 8.5 | ± | 2.4* | 25 | ± | 25*^†‡§^ |
| I/R+T3 (300 µg/L)+RISK-BL | baseline | 13.6 | ± | 1.7 | 102 | ± | 16 |
| (n=9) | isch5 | 0.0 | ± | 0.0* | 1 | ± | 1* |
|  | isch25 | 0.0 | ± | 0.0* | 3 | ± | 3* |
|  | rep10 | 8.6 | ± | 1.5* | 12 | ± | 14*^‡§^ |
|  | rep30 | 9.0 | ± | 1.5* | 21 | ± | 18*^#†‡§^ |
|  | rep60 | 8.6 | ± | 1.2* | 24 | ± | 16*^†‡§^ |
| I/R+T3 (300 µg/L)+SAFE-BL | baseline | 13.8 | ± | 1.7 | 111 | ± | 15 |
| (n=10) | isch5 | 0.0 | ± | 0.0* | 1 | ± | 1* |
|  | isch25 | 0.0 | ± | 0.0* | 2 | ± | 3* |
|  | rep10 | 8.1 | ± | 2.5* | 32 | ± | 24* |
|  | rep30 | 9.2 | ± | 2.3* | 57 | ± | 25* |
|  | rep60 | 8.6 | ± | 2.1* | 47 | ± | 21* |

Values are means±standard deviations. n: number of rats; baseline: last min of stabilization period before ischemia/ reperfusion; isch5/25: 5/25 min of ischemia; rep 10/30/60: 10/30/60 min reperfusion; CF: coronary flow; I/R: ischemia/reperfusion; ERK-BL: blockade of mitogen extracellular-regulated kinase 1/2; iPoCo: ischemic post-conditioning; LVDP: left ventricular developed pressure; PI3K-BL: blockade of phosphatidylinositol(4,5)-bisphoosphate-3-kinase; RISK-BL: blockade of the reperfusion injury salvage kinase pathway; SAFE-BL: blockade of the survival activating factor enhancement pathway; TMP: time-matched perfusion without I/R; T3: addition of triiodothyronine 100, 200, 300 or 500 µg/L at reperfusion. Baseline values for CF and LVDP were analyzed by one-way ANOVA and Fisher’s least significant difference post-hoc tests. Time courses for CF and LVDP were analyzed by two-way ANOVA for repeated measures and Fisher’s least significant difference post-hoc tests between the groups I/R, I/R+iPoCo, I/R+T3 (100 µg/L), I/R+T3 (200 µg/L), I/R+T3 (300 µg/L), I/R+T3 (500 µg/L); *p<0.001 vs. baseline, respectively; †p<0.05 vs. I/R+iPoCo; (†)p=0.060 vs. I/R+iPoCo; ‡p<0.05 vs. I/R+T3 (100 µg/L); §p<0.05 vs. I/R+T3 (500 µg/L). Time courses for CF and LVDP in blocker experiments were analyzed by two-way ANOVA for repeated measures and Fisher’s least significant difference post-hoc tests between the groups I/R, I/R+iPoCo, +I/R+T3 (300 µg/L), I/R+T3 +(300 µg/L)+PI3K-BL, I/R+T3 (300 µg/L)+ERK-BL, I/R+T3 (300 µg/L)+RISK-BL; I/R+T3 (300 µg/L)+SAFE-BL; **p*<0.001 vs. baseline, respectively; #p<0.05 vs. I/R; ‡p<0.05 vs. I/R+SAFE-BL; †*p*<0.05 vs. I/R+T3 (300 µg/L); §p<0.05 vs I/R+T3 (300 µg/L)+SAFE-BL.
